# Ocular complications in pediatric non-infectious anterior uveitis in long-term follow-up: population-based cohort study

**DOI:** 10.1186/s12348-025-00499-1

**Published:** 2025-05-06

**Authors:** Mira Siiskonen, Roosa Pesälä, Pasi Ohtonen, Anna-Maria Kubin, Nina Hautala

**Affiliations:** 1 Department of Ophthalmology, Research Unit of Clinical Medicine and Medical Research Center, University of Oulu, Oulu, Finland; 2 Oulu University Hospital, Oulu, Finland; 3 Research Service Unit, Oulu University Hospital, Oulu, Finland; 4 The Research Unit of Surgery, Anesthesia and Intensive care, Oulu University Hospital, University of Oulu, Oulu, Finland; 5 Department of Ophthalmology, University of Oulu, Oulu University Hospital and MRC Oulu, P.O.Box 21, Oulu, OYS 90029 Finland

**Keywords:** Pediatric uveitis, Ocular complications, Juvenile idiopathic arthritis, Idiopathic uveitis

## Abstract

**Background:**

Pediatric uveitis is often asymptomatic, which may expose to sight-threatening ocular complications. The impact of modern medication on frequency of long-term ocular complications in pediatric patients with anterior idiopathic uveitis (idio-U) or juvenile idiopathic arthritis associated uveitis (JIA-U) is not fully understood. We aimed to evaluate the occurrence of ocular complications during the era of modern treatment on the population-based cohort of children with idio-U or JIA-U.

**Methods:**

A longitudinal, population-based cohort study of children with idio-U or JIA-U in 2008–2020. Variables assessed included age, gender, age at diagnosis, laterality, chronicity, vision, and ocular complications.

**Results:**

107 pediatric patients and 172 eyes with either idio-U (19 patients) or JIA-U (88 patients) were included. The mean age at uveitis onset was 10.0 ± 3.7 and 5.4 ± 3.2 years in idio-U and JIA-U, respectively (*p* < 0.001). Uveitis was chronic in 58% in idio-U and 74% in JIA-U patients, respectively. Uveitis was complicated with glaucoma in 45% of idio-U and 18% of JIA-U patients (*p* = 0.019). Cataract was developed in 31% of idio-U and 22% of JIA-U eyes (*p* = 0.28), and posterior synechiae in 21% and 9% of the eyes with idio-U and JIA-U, respectively. None of the eyes were hypotonic. Female gender was overrepresented in ocular complications. Glaucoma surgery was accomplished in 25 (15%) and cataract surgery in 19 (11%) eyes. Bilateral visual acuity remained > 0.5 in all patients.

**Conclusions:**

Glaucoma, ocular hypertension, and cataract were the most typical complications of uveitis. Complications occurred mostly in girls and in idio-U patients. JIA-U patients with severe uveitis, young age at uveitis onset and female gender were predisposing factors for surgical management.

## Background

Uveitis in children is rare, representing only 5–10% of all cases of uveitis [1, 2]. Among the known risk factors for uveitis in children with rheumatologic diseases are female gender, age under 7 years at onset, oligoarticular subtype, and ANA positivity [2]. Typical asymptomatic nature of pediatric uveitis may cause delays in the diagnosis of ocular inflammation and initiation of treatment predisposing the development of serious sight-threatening complications, including cataract, glaucoma, macular edema, and ocular hypotony [1, 3, 4]. Effective screening for uveitis in patients with juvenile idiopathic arthritis (JIA) has enhanced early diagnosis of JIA-associated uveitis (JIA-U) [2]. Idiopathic uveitis (idio-U) in children is commonly diagnosed later, and the delayed treatment of ocular inflammation may predispose for development of ocular complications.

Risk factors for the severity of juvenile idiopathic arthritis (JIA)- associated uveitis include male sex, young age at onset of JIA (< 7 years), uveitis severity at presentation, short (< 6 months) interval between JIA and uveitis onset, and presence of synechiae at first visit [5–7]. JIA onset after the age of 5 years, no use of corticosteroids, and use of adalimumab treatment has been shown to be associated with uveitis inactivity for at least 6 months [8]. Modern treatment modalities of uveitis have improved the visual prognosis of pediatric uveitis [8–10]. The long-term impact of modern medications including disease-modifying antirheumatic drugs (DMARDs) and biologic therapy (bDMARDs) on frequency and severity of uveitis complications in children is not fully established.

The aim of this study is to evaluate the features of ocular complications in pediatric uveitis during the long-term follow-up period of 2008–2020. The presence and severity of uveitis complications is studied in a population-based cohort of children with either idio-U or JIA-U.

## Main text

### Methods

This retrospective interventional case series was performed at Oulu University Hospital. The study followed the tenets of the Declaration of Helsinki, and it was conducted with the approval of the Oulu University Hospital Research Committee (89/2017). It was not appropriate to involve patients or the public in the design, or conduct, or reporting, or dissemination plans of the study.

The population-based cohort consisted of pediatric patients < 16 years of age who presented at Oulu University Hospital with idio-U or JIA-U between Jan 1, 2008, and December 31, 2017. All patients were followed up until March 31, 2020. The treatment of pediatric uveitis was completed at Oulu University Hospital responsible for tertiary care for a population of approximately 410 000 inhabitants. The hospital’s electronic patient database was used to search for patients with uveitis by using the ICD-10 (International Classification of Diseases) diagnosis codes for anterior uveitis (H20.0 and H20.1). Patients with uveitis other than idio-U or JIA-U were excluded. Data included parameters for age, gender, age at uveitis onset, laterality, chronicity, anatomical distribution of uveitis, best-corrected visual acuity (BCVA), and intraocular pressure (IOP). Data from ocular complications (glaucoma, ocular hypertension > 30 mmHg, ocular hypertension > 21 mmHg, ocular hypotony, cataract, posterior synechiae, band keratopathy, papilla and macular edema) were gathered at the end of follow-up. Uveitis classification was based on the criteria of the Standardization of Uveitis Nomenclature (SUN) [11]. JIA was diagnosed in children according to the International League of Associations for Rheumatology Classification [12] and screening for JIA-U was carried out according to the screening guidelines described earlier by Heiligenhaus et al. [2]. Diagnosis of secondary glaucoma was based on elevated IOP and optic nerve rim loss. Patients who experience an IOP rise due to glucocorticoid use are called corticosteroid responders (moderate rise > 10 mmHg from baseline). Visual acuity was evaluated by the Snellen chart, and the affected eye or the eye with more uveitis activity and/or worse BCVA in cases of bilateral uveitis was analyzed. The WHO criteria of distance vision impairment (Snellen visual acuity < 0.3 [6/18] or < 59 ETDRS letters in a better eye) was used.

The treatment and follow-up of uveitis was based on the treatment guidelines of uveitis for pediatric patients. Briefly, topical glucocorticoids are the first-line treatment for children with anterior uveitis. Methotrexate treatment was initiated if the requirement for topical glucocorticoids exeeded two drops per day after one month of treatment. If patients exhibited no response to methotrexate within three months, or if they experienced ocular hypertension (IOP > 25 mmHg) or other complications of uveitis such as macular oedema, optic disc swelling or epiretinal membranes, adalimumab was included in the treatment. Dexamethasone implants were inserted for diagnosed macular oedema and to prevent postoperative inflammation after cataract surgery in high-risk patients, as deemed necessary.

Summary data are presented as mean with standard deviation (SD) for the continuous variables. Categorical data are expressed as frequencies with percentages. When comparing data at patient level the Student’s t-test or the Welch test was used for between group comparisons for continuous data and The chi-square test or the Fisher’s test was used to evaluate the differences between categorical data.

Several patients had bilateral uveitis diagnosis. When comparing data at the eye level the Linear Mixed Model (LMM) for continuous data or Generalized Linear Mixed Model (GLMM) for categorical data was used for analyses. Patient was set as a random effect in both LMM and GLMM to take account the dependency between eyes within a patient.

Statistical significance was set at p value < 0.05. The SPSS for Windows (IBM Corp. Released 2021. IBM SPSS Statistics for Windows, Version 28.0. Armonk, NY: IBM Corp) and SAS for Windows (version 9.4 SAS Institute Inc., Cary, NC, USA) were used to perform all statistical evaluations.

## Results

A total of 107 pediatric patients and 172 eyes with either idio-U (19 patients, 29 eyes) or JIA-U (88 patients, 143 eyes) were included in the present study. From the total of 107 patients, 65 (61%) were girls. Most patients, 58 (89%) of the girls and 30 (71%) of the boys had JIA-U (*p* = 0.036). The mean age at uveitis onset was 10.0 ± 3.7 and 5.4 ± 3.2 years in idio-U and JIA-U, respectively (*p* < 0.001). Most patients, 58% in idio-U and 74% in JIA-U, had chronic anterior uveitis, which was bilateral in 53% and 63% of idio-U or JIA-U patients, respectively. Among patients with idio-U, 10 (53%) and 49 (56%) patients in JIA-U, were corticosteroid responders. Patients were followed up for 101.4 months on average (range 1-250 months). Demographic data is presented in Table 1.

**Table 1:** Demographic characteristics of study participants

	Total	idio-U	JIA-U	*P* value
Patients, n (%)	107	19 (18)	88 (82)	
Female gender, n (%)	65 (61)	7 (37)	58 (66)	0.036
Corticosteroid responder, n (%)	59 (55)	10 (53)	49 (56)	0.80
Bilateral	65 (61)	10 (53)	55 (63)	0.46
Eyes, n (%)	172	29 (17)	143 (83)	
Mean age at first uveitis, months (SD) median	101.4 (57.2) 98.2	75.2 (51.7) 65.2	106.8 (56.9) 100.5	< 0.001
Chronic	76 (71)	11 (58)	65 (74)	0.17

At the end of the follow-up period, uveitis was complicated with glaucoma in a total of 39 (23%) of all eyes: 13 (45%) eyes in idio-U and 26 (18%) in JIA-U (*p* = 0.019) (Table 2). Ocular hypertension > 30 mmHg was noted in 13 (45%) eyes in idio-U and 35 (24%) in JIA-U (*p* = 0.087). Intraocular pressure (IOP) > 21 mmHg occurred in 17 (59%) in idio-U and 39 (27%) in JIA-U (*p* = 0.019). Ocular hypotony did not exist in a study cohort. Cataract developed in a total of 40 (23%) of the study eyes. Nine (31%) eyes in idio-U and 31 (22%) in JIA-U had cataract. Posterior synechiae was diagnosed in 19 (11%), band keratopathy in 9 (5%), optic disc swelling in 6 (4%), and macular edema in 5 (3%) of all study eyes.

**Table 2:** Complications of anterior idiopathic uveitis (idio-U) and juvenile idiopathic arthritis associated uveitis (JIA-U) in children

	Total	idio-U	JIA-U	*P* value	95% Confidence Interval
Eyes, n (%)	172	29 (17)	143 (83)		
Visual acuity Snellen charts, mean (SD)		1.00 (0.25)	0.99 (0.26)	0.84	-0.01^1^ -0.13 to 0.11
Glaucoma, n (%)	39 (23)	13 (45)	26 (18)	0.019	4.19^2^ 1.27 to 13.9
Ocular hypertension > 30, n (%)	48 (28)	13 (45)	35 (24)	0.087	2.77^2^ 0.86 to 8.87
Ocular hypertension > 21, n (%)	56 (33)	17 (59)	39 (27)	0.019	4.44^2^ 1.28 to 15.4
Hypotony	0 (0)	0 (0)	0 (0)	n.d.	n.d.
Cataract, n (%)	40 (23)	9 (31)	31 (22)	0.28	1.91^2^ 0.58 to 6.28
Posterior synechiae, n (%)	19 (11)	6 (21)	13 (9)	0.16	2.66^2^ 0.67 to 10.6
Bandkeratopathy, n (%)	9 (5)	2 (7)	7 (5)	0.66	1.44^2^ 0.27 to 7.62
Optic disc swelling, n (%)	6 (3.5)	1 (3.4)	5 (3.5)	0.99	1.01^2^ 0.09 to 10.9
Macular oedema, n (%)	5 (2.9)	0 (0)	5 (3.5)	0.59	n.d.

^1^ Difference between means

^2^ Odds ratio

^n.d^. not definable

Female gender was overrepresented in ocular complications: 73% of patients with glaucoma, 71% with ocular hypertension, and 52% of those with cataract were girls. The mean age of uveitis onset differed significantly in patients with or without ocular hypertension > 21 mmHg, 6.4 years vs. 5.6 years, respectively (*p* = 0.017). Age at uveitis onset was 6.2 vs. 5.8 years with or without ocular hypertension > 30 mmHg (*p* = 0.14), and 6.2 vs. 5.8 years for patients with or without cataract (*p* = 0.19).

Long-term visual outcomes were significantly affected by ocular complications of uveitis. An average BCVA was 0.8 ± 0.04 vs. 1.0 ± 0.02 in children with or without glaucoma (*p* < 0.001), 0.9 ± 0.04 vs. 1.0 ± 0.03 in those with or without ocular hypertension > 21 mmHg (*p* = 0.003), and 0.9 ± 0.04 vs. 1.0 ± 0.03 in patients with or without ocular hypertension > 30 mmHg (*p* < 0.001), respectively.

Glaucoma surgery was completed in 3 eyes with idio-U and 22 eyes with JIA-U consisting of implantation of Molteno or Baerweldt or deep sclerectomy. Cyclophotocoagulation was accomplished in 2 patients with JIA-U. Some of the patients had several glaucoma operations during the follow-up time and 7 children with JIA-U had glaucoma operated in both eyes. A total of 19 eyes underwent cataract surgery (3 idio-U and 10 JIA-U patients). 6 children had cataract extraction in both eyes, they all were JIA-U patients. One patient with JIA-U had corneal transplantation and 2 corneal peeling (EDTA chelation) during the follow-up. From all patients who had glaucoma surgery or cataract extraction, 72% and 54% were girls, respectively.

Eight patients, 1 with idio-U and 7 with JIA-U, had permanent decline in BCVA < 0.5 at the end of the follow-up in the worse eye, but none in both eyes. Two of these patients had BCVA < 0.5 already at the onset of uveitis. The average age of uveitis onset in these patients was 4.8 years, and 50% were girls. The patients with decreased BCVA had several simultaneous ocular complications: glaucoma in 4 (50%) of the patients, ocular hypertension > 21 mmHg in 4 (50%), ocular hypertension > 30 mmHg in 4 (50%), cataract in 7 (88%), band keratopathy in 3 (38%), macular edema in 3 (38%), and optic disc swelling in 1 (13%). Five (63%) out of 8 patients with irreversible decline in unilateral BCVA had bilateral uveitis, 4 (50%) were corticosteroid responders, 2 (25%) were using biologic therapy and 3 (38%) were treated with methotrexate.

## Discussion

In this retrospective population-based cohort study, we found that pediatric patients with idio-U have more uveitis complications after long-term follow-up compared to children with JIA-U, which is in line with the previous study [13, 14]. Especially, the rates for glaucoma and ocular hypertension were significantly higher in idio-U vs. JIA-U. Increased rate for ocular complications was related to older age at the onset of uveitis (10.0 years in idio-U vs. 5.4 years in JIA-U), and ocular complications in idio-U patients may occur already at the onset of uveitis. The asymptomatic nature of idio-U prior to development of ocular complications and the absence of screening may lead to delayed diagnosis of uveitis and thus explain the increased risk of ocular complications in patients with idio-U. In addition, treatment of uveitis with DMARDs and bDMARDs known to improve the prognosis of uveitis is initiated typically later in patients with idio-U than those with JIA-U.

Previous study has revealed ocular complications in 67% of the children with JIA-U evaluated in 1984–2005 [6]. In another study from India, 66% of children evaluated in 2009–2020 had ocular complications of uveitis during the follow-up [15]. These numbers are much bigger than noted in our population-based cohort. One explanation for this discrepancy might be the implementation of modern and more efficient treatment of uveitis especially for children with JIA-U in Finland during the last two decades [9]. Female gender was over-represented in ocular complications among the participants of the present study: 73% of patients with glaucoma, 71% with ocular hypertension, and 52% of those with cataract were girls. This differs from previous studies that revealed that male sex is associated with more severe uveitis and poorer prognosis [5, 7].

A combination of inflammation and steroid therapy led to development of glaucoma in 23% of the eyes in the current study. Recent studies have shown that 26–30% of the pediatric patients with uveitis developed glaucoma [16, 17]. It was more prevalent in idio-U than in JIA-U in the present cohort; 45% of the eyes with idio-U and 18% with JIA-U developed glaucoma. In contrast to this, Cann et al. suggested recently that children with JIA-U were more likely to develop glaucoma [18]. Ocular hypertension over 30 mmHg was noted in 28% of the eyes in the current cohort, which is equal compared to 29–36% of patients with uveitis and ocular hypertension in recent studies [19, 20]. It is also notable, that 55% of all children in this study cohort were corticosteroid responders, which is much greater compared to approximately 20% in the healthy population predisposing the increased risk for glaucoma. In the present cohort, none of the patients developed ocular hypotony in a long-term follow-up. In contrast, 16% prevalence of ocular hypotony has been reported previously in patients with JIA-U [21]. Hypotony is a well-recognized, sight-threatening complication of uveitis, which may lead to development of phthisis bulbi and be the final endpoint for a multitude of disease entities. Long duration of uveitis, low BCVA, high anterior chamber flare, and the presence of posterior synechiae at the onset of uveitis are among the factors predicting the risk for ocular hypotony [21].

Uveitic cataract has been shown to be one of the most common complications of pediatric uveitis, and it may occur secondary to a combination of inflammation, steroid therapy, or posterior synechiae [6, 15, 18, 22]. Cataract was noted in 19–21% of the patients with pediatric uveitis in the recent studies [17, 19, 20, 23]. Surgical treatment of pediatric uveitis related cataract has been shown to be effective and relatively safe intervention, which improves the visual outcomes of children with uveitis [24, 25].

Although ocular complications were more common in idio-U than JIA-U, surgical treatment was more often needed in patients with JIA-U than in idio-U. This might be explained by exceptional severity of uveitis associated with younger age at uveitis onset in JIA-U patients, as reported previously [7]. In agreement with this, the average age of uveitis onset was only 4.8 years in study patients with permanently decreased BCVA < 0.5 in the worse eye. Despite the statistically significant differences in BCVA in patients with or without ocular complications, long-term vision remained good in all study participants. In agreement with our results, AlBloushi et al. has reported that a majority, 93%, of pediatric patients with uveitis have BCVA ≥ 0.5 with quiescent uveitis [20]. The 18% incidence of macular edema has been recently reported in patients with JIA-U and it is one of the main causes for decreased BCVA in children with uveitis [3, 18]. The prevalence of macular edema was only 3% in the current cohort which may at least partly explain the good visual outcomes of the participants especially when treated with intensive immunomodulatory therapy [9].

As a retrospective study we acknowledge certain limitations to the study. For example, in addition to clinical examination including biomicroscopy no certain classification for cataract severity was used. Diagnostic evaluations for glaucoma might have varied according to the age of the patient examined, and comprehensive fundus imaging are lacking in some patients. However, a long follow-up and a fully covered population-based cohort of children with idio-U or JIA-U can be considered as strengths of the study, although further studies in larger populations are needed to fully elucidate the impact of modern treatment on the occurrence and prognosis of ocular complications of pediatric uveitis.

The presence of ocular complications leads to requirement for recurrent ophthalmic examinations and commitment to long-term treatment, which may have a negative effect on health-related quality of life (HRQoL) in children with uveitis [26]. Early diagnosis of uveitis, its adequate treatment with modern immunomodulatory therapy and efficient management of ocular complications influence the visual outcome in children with uveitis, regardless of the etiology of the disease. Moreover, stability or improvement of visual function despite uveitis complications have been shown to improve HRQoL [27]. The visual prognosis of uveitis in children has improved via implementation of modern medications, but there is still potential for further developments in timely diagnosis and treatment outcomes in the future.

## Conclusions

Glaucoma, ocular hypertension, and cataract were the most common ocular complications in pediatric uveitis, and they occurred mostly in girls and in idio-U patients. JIA-U patients with severe uveitis, early uveitis onset and female gender predisposed for surgical management. Despite improved visual prognosis of pediatric uveitis via implementation of modern medications, further efforts for earlier diagnosis of uveitis to prevent ocular complications are needed.

## Data Availability

No datasets were generated or analysed during the current study.

## Abbreviations

ANA: Antinuclear antibody
BCVA: Best corrected visual acuity
bDMARDs: Biologic disease-modifying antirheumatic drugs
DMARDs: Disease-modifying antirheumatic drugs
GLMM: Generalized Linear Mixed Model
HRQoL: Health-related quality of life
ICD-10: International Classification of Diseases
idio-U: Anterior idiopathic uveitis
IOP: Intraocular pressure
JIA: Juvenile idiopathic arthritis
JIA-U: Juvenile idiopathic arthritis associated uveitis
LMM: Linear Mixed Model
SUN: Standardization of Uveitis Nomenclature
